# Understanding the Person through Narrative

**DOI:** 10.1155/2011/293837

**Published:** 2011-05-04

**Authors:** Joanne M. Hall, Jill Powell

**Affiliations:** ^1^College of Nursing, University of Tennessee, 1200 Volunteer Boulevard, Knoxville, TN 37996, USA; ^2^BeardenPsych, 5401 Kingston Pike, Knoxville, TN 37919, USA

## Abstract

Mental health nurses need to know their clients at depth, and to comprehend their social contexts in order to provide holistic care. Knowing persons through their stories, narratives they tell, provides contextual detail and person-revealing characteristics that make them individuals. Narratives are an everyday means of communicating experience, and there is a place for storytelling in nearly all cultures. Thus narrative is a culturally congruent way to ascertain and understand experiences. This means the nurse should ask questions such as “How did that come about?” versus why questions. A narrative approach stands in contrast to a yes/no algorithmic process in conversing with clients. Eliciting stories illustrates the social context of events, and implicitly provides answers to questions of feeling and meaning. Here we include background on narrative, insights from narrative research, and clinical wisdom in explaining how narratively understanding the person can improve mental health nursing services. Implications for theory, practice, and research are discussed.

## 1. Understanding the Person through Narrative

Asking “What is your story? “will provide more knowledge about persons than asking “How are you?” Mental health nurses want to provide holistic care with in depth knowledge of persons in the context of their daily lives. How can we as nurses deeply know individuals without knowing their particular stories? These are the questions being considered in steadily increasing narrative-based health research [1–6]. New insights from this research as well as the principles of narrative can inform mental health nursing practice, because stories are the form of language most often used to convey experiences.

## 2. Narratives and Nursing

Narratives have been central to nursing and will continue to be so. Nursing has a long history of using narratives in the form of clinical stories in communicating about patients, and in relating information among practitioners [7–10]. The patient history is based in part on narrative information as context for specific findings from examination [11, 12]. Thus narrative has relevance to nursing practice on several levels.

Mental health is a field of nursing practice that might benefit from an even greater understanding and use of narrative in specific subpopulations, and at transitional points in the individual person's life. Examples of transitional points are times of crisis, developmental milestones, relationship changes, getting a psychiatric diagnosis, and exacerbations. As persons, we use narrative to frame everyday events as well as disruptive or unexpected life changes, whether these events are physiological, behavioral, and/or social [13–17]. Concerning a mental health client whose illness is newly emergent or is exacerbated, nurses pay attention to, but might not name, what they see as a disruption in the life story. Therefore, examining narratives more explicitly as a way to frame mental health practices is potentially enhancing of patients' recovery, quality of life, and occupational success. Moreover, the transfer of knowledge among nurses via clinical narratives could be even more explicit, providing a way for novices to hone their situation-specific skills and clinical wisdom. This is especially true in mental health, where much of our care is focused on improving clients' physical and social well-being when experiencing a psychiatric disorder, and minimizing the social disruption that this can cause. 

Narrative is also a way that patients relate and integrate past events, such as prior interpersonal trauma or neglect [5, 14, 18, 19] that are often intertwined with current symptoms. Culturally relevant information, such as that pertinent to African Americans, for example, is embedded in the stories clients tell, making for more holistic, culturally sensitive, contextualized knowledge of clients, their families, and support networks [20–24],

## 3. Purpose Statement

The purpose of this paper is to provide basic conceptual information about narratives, and to provide useful approaches to mental health nursing care when the client is understood from a narrative perspective. Background information about narratives is included as well as exemplars that illustrate these points so that even novice nurses might use a narrative framework for learning about and caring for psychiatric patients. Narratives reveal the complexities of relating to others as well as the outcomes of such interaction. This is not a research report, but some realistic exemplars will facilitate adopting narrative approaches to care. As noted, some examples come from unpublished data in a recent narrative study [5, 6]. Later exemplars are derived from actual clinical interactions from the second author's prescriptive practice. These quotations are edited for conciseness, and identifiers have been removed.

## 4. What Are Narratives?

Polkinghorne emphasizes that narratives are communicative of events and that the form/structure of narratives links the parts together in a meaningful, coherent whole [16]. Riessman has a broader definition, simply holding that narrative is talk about events that are consequential, events that are temporal though not necessarily told chronologically, having meaning for the teller and listener [1]. Bruner emphasizes the cultural nature of narratives, in which shared expectations and beliefs are communicated, so that narratives communicate wider social meanings. 

Based on these tenets, narratives are defined here as means of communication that reflects time-ordered events with a discernible plot and cast of characters and that imparts personal and cultural information from the teller to the audience in a coherent whole. The series of events in a story may be told with a different meaning or purpose in each telling. Both structure and content of narratives can be examined for differences and commonalities among similarly situated individuals. Narratives need not be conveyed in verbal language, but are evident also in music, visual images, and symbols [25]. 

Bruner also emphasizes the constructed nature of narratives, holding that they represent the pulling together of parts of a self that is then known within an environment that is subjectively experienced [13]. From this viewpoint, stories can be considered as carrying themes, or “selves” that make up a larger, overarching self-narrative. 

 Levels of narrative are differentiated by Bal as the fabula, or series of events on which a story is based, story, referring to the manner of telling these events, and narrative text, which is created when the narrative is set forth in words, music, or images [25, 26]. In this paper, “narrative” is used interchangeably with “story,” with the understanding that narratologists vary in differentiating these terms. This is in part due to the interdisciplinary nature of narratology, having originated in the study of linguistic aspects of fiction, such as the novel. A full explanation of these distinctions is beyond the scope of this paper [25, 27–29]. A caveat is that in some cultural settings, the term story might best be avoided because it can connote deception. For example, in the southern U.S. “telling a story” refers to lying. That is not the case in this paper. Conversely, even “lies” can hold narrative truth, as we shall explain.

### 4.1. Narrative Truth

Although there has been debate as to the verifiability of stories told in the context of mental health treatment, especially those about childhood trauma in an adult's past, as in the case of potential “false memories,” (e.g., [30]). The preponderance of the research shows that these are rare (e.g., [31–33]). Most stories of past trauma told by clients possess narrative truths, even if details are in error [1, 34–37]. Narrative truth refers to the phenomenon of having the core plot and meaning of a story subjectively consistent for the person telling it, while the actual historical events may differ; narrative approaches to the nature of subjective experience allow for multiple realities [38, 39]. Paley and Eva caution against romanticizing personal narratives, stating that there is no inherent authenticity about them, suggesting that caregivers be vigilant about how to use narratives as part of a history [40]. What is clear is that narratives cannot definitively reveal historical facts, but rather the meanings, life patterns, and emotional impact events have for clients.

The mental health nurse is most interested in the client's subjective experience, and in this case, absolute certainty about how events have occurred for the client, but rather how they are related. According to Bruner, narratives are always being reconstructed, and reveal what is salient in experiences for clients telling their stories, and what they want others to know about their experience. The constructed narrative shows events that are part of the client's world and perceptions about these events even referring to the future world in which the teller aims toward creating [13, 41]. Narrative truth is a term that applies to the consistency of general themes revealed by a narrative, especially its core theme. Even an obviously invented narrative, for example, can be a metaphor for boundary violations, abandonment, betrayal, and other disturbances that the person might be trying to communicate [42].

Stigma is associated with psychiatric diagnoses and upon being so-labeled, clients often stubbornly resist, and this resistance may be central to the daily problems they experience in the course of their illness [43]. Roe and Kravetz say:

“Many people seem unable to reap the benefits of accepting a psychiatric diagnostic label without having to constantly sow the sorrows that frequently are associated with this acceptance. This feeling raises the question of whether there are conditions and processes that can enable us to foster the beneficial aspects of being aware of and acknowledging a psychiatric disability without incurring the risk of the personal and social harm sometimes produced by such awareness and acknowledgement” (p. 418).

These authors stress that putting experiences of diagnosis and the illness itself into a narrative is helpful. Narratives of “care” involving diagnostic labeling, with the concomitant engulfing of self-stigmatization that often occurs, even when the psychiatric diagnosis is accurate, may still be harmful [43]. Furthermore, in terms of narrative truth, even in the face of telling a story imprecisely, “Narrative facts may represent attempts to communicate the specific emotional experiences...to gain control over these events, and to gain consolation and joy from transforming these events into a story (p. 421)” [43]. One goal of narrative then is to gain mastery or control in trying circumstances, often including difficult interactions with mental health providers.

Wilkinson and Hough [42] used the word “lies” to mean “unbelievable stories that our patients insisted we believe unquestioningly” (p. 581). For example, teens who were adopted, as a result of being neglected and maltreated by the parents of origin, constructed amazing tales in which they achieved revenge for or control over abuse. In their stories, the narrator/self/client appears strong and resists abandonment themes. Narrative truth in these cases lies in the form and themes of the story, and the role the narrator/client occupies in the story, versus how s/he *wants* to be (as in one client's unbelievable account of rescuing others from all kinds of catastrophes). If the therapist can use countertransference to inform an understanding of the treatment process, an appreciation emerges that the “truth of the lie is in its impact” (p. 580). Decisions about how to intervene thus depend on the client's subjectivity, a matter of narrative truth, not historical fact.

### 4.2. Narratives Are Coconstructed

Narratives are co-constructed between teller and listener, writer and reader, who together create the story because each brings a particular standpoint to the story. In addition to revealing subjective experiences, caregivers should consider the version of the story that is told to a particular “audience” for specific purposes [1, 2, 25]. Stories may reveal personal, family, or larger collectives including goals, ideals, and cultural beliefs. Personal stories include remembered chronologies of events; identification stories signifying gender, race, class, partner status, and cultural and political implications of identities based on these aspects of self [13]. Hence, narratives are a means to reveal life context, the time and space environment experienced by the client, including sociopolitical dynamics.

## 5. Types of Narratives Relevant to Mental Health

Narratives are told in specific settings, taking forms in which pertinent kinds of information is made available to mental health nurses. We often hear these stories, but perhaps do not stop for at least a brief analysis of what and why we heard a particular kind of story. Some relevant types are discussed here.

### 5.1. Self Narratives

Crucial to mental health nursing is the self/life narrative. It has been posited that in a very substantive way we *are* our stories [13]. Frank says that stories “contain” the self [44]. Stories reveal aspects of the self in the context of one's history and the likely future trajectory. Development is understood in a new and contextualized way through the use of narratives [20, 45] Stories are dependent on abilities to remember, and memories making up the totality of “self” are stored as narratives [2, 29, 35, 36, 46]. The lack of a consistent, continuous life narrative that occurs when memory is disturbed is evident in the person with advanced Alzheimer's disease, for example, with all of the social disruption and suffering it causes. A self-narrative may be of a discrete episode about belongingness, for example, or it could be a lengthy life history, told chronologically from earliest experiences forward to the present.

Often, the family of origin stories we tell and retell signify key family roles and dynamics that have an impact on the construction of the self, [47]. Here again we are aware that some of the actual facts of these stories cannot be precisely known. Changes, memory gaps, secrecy, the identity of the given audience, and the focus of emphasis make for different tellings. These changes may also be influenced by phases of development, current situations, and the power status of the players or characters in the story. The manner in which family stories are constructed and reconstructed, also bring insight about the characteristics of actual persons about whom the story is told. Future narratives about one's family reveal goals, expectations, and anticipated, hoped-for changes.

### 5.2. Illness Narratives

The psychiatric client has a number of stories to tell about health and sickness. Some have to do with mental illness itself. Key stories are of childhood, symptoms experienced, responses to being given a psychiatric diagnosis, work experiences, social support, and so forth. Frank discusses three genres of health narratives: (a) technoluxe (concerning highly advanced techniques affordable to a few), (b) unbearable (care denied or not accessible), and (c) strategic [48]. In mental health we often hear the unbearable stories, but perhaps might have to dig harder to get to the clients patterns of strategic behavior they use in response to psychiatric symptoms. Frank says patients have a need to hear their own voices as happens in the telling of stories. He also identifies five dramas or stories of conflict relative to illness: (a) genesis (how the illness is thought to have occurred), (b) emotion work (what feelings can be displayed, often “altered” by the patient to protect others, such as family, from the realities of illness), (c) fear and loss (anticipating or wondering how bad it can get, common in mental health), (d) the drama of meaning (whether or not one can continue with a story that precipitates more problems), and (e) dramas of self (who must heroically transcend the fear and losses sustained). He adds that stories “take care of people by affirming...[and] what is affirmed is less important than the act of affirmation” (p. 389) [44]. He sums up the power of narrative in illness.

What makes narrative powerful is its singular ability to be both comforting and dangerous. What is culturally essential about narrative is its singular capacity to remind us that both comfort and danger, joy and plight, not only can be reconciled, but may actually require one another” (p. 380).

Also to be considered is that psychiatric clients also have some ongoing “physical” problems, especially as the aging process occurs. These are important narratives, and very useful. For instance, the fatigue from cancer, and cancer treatment, can be confused for, but yet also sometimes accompanies, depression. How the client is positioned in terms of treatment and recovery from the physiological problem(s) encumbers and changes the focus of mental health treatment. These are illness stories we must also hear if treatment is to be integrated, consistent with holism.

Illness narratives thus constitute a distinct type [1, 49–51], including stories of psychiatric illness [14]. People form their own explanations about the causes of their illnesses. In some cases a particular genre of story is evident, such as victory, struggle with adversity, decline, or loss. In addition, the identity is painted according to the effects illness has had and changes that become necessary in order to go on with life. Frequently, illness narratives are more about the social suffering (versus the physiological suffering) experienced by the ill or disabled person, including a loss of power or agency as in the imbalance of a relationship in which the caregiver is seen as a powerful gatekeeper [1].

Surviving trauma and stories of recovery from trauma are a specific form of narrative similar to illness narratives, in that hurdles overcome and lingering effects of the trauma coexist with evidence of coping [5, 18, 52]. A stigmatized individual, such as someone with a known mental illness may embrace a story that plays against being defined as “other.” These are called resistance narratives, and sometimes one person's story of resistance is a kind of testimony, iconic of others with the same or similar set of obstacles overcome [38].

### 5.3. Trauma Narratives

Clearly, many psychiatric clients have suffered previous trauma, and because of vulnerabilities, they may be currently experiencing trauma, especially in the form of interpersonal violence. Trauma narratives may refer to recent or distant events. Flashbacks are most common in narratives of recent events [53]. Rather than asking yes or no questions about interpersonal trauma, such as childhood maltreatment, providers might instead ask “What do you recall happening when you were young? Were there hard times?” This phrasing was found to be helpful in a recent narrative study of thriving after abuse, in which admitting to having been “abused” was avoided by the (mostly Appalachian) participants, but many of the lines of evidence of abuse were elicited when questions were framed as hardship stories of early life [5].

The aforementioned study reinforced knowledge about trauma narratives. Here, some unpublished statements in the data are illustrative. Trauma narratives were sometimes long and detailed, or conversely, some were truncated, even to a single sentence, such as “My uncle raped me, and that is all I can say about it.” One theory about this is that successful integration of the traumatic experience enables the victim to speak at length in a narrative about abuse, for example [34]. Sometimes metaphors are powerful in revealing the impact of events [51], as in “I was on a sinking ship and there was no way out”. Turning points often emerge in stories, sometimes in change of tense or evaluative remarks as in the following: “I told him I would report him to the police. Just then I realized he was actually scared of me. So I am not anxious or afraid now when I see him at a family gathering or on the street.” [5].

Along with narratives of victimhood, caregivers may also elicit narratives of resilience or even thriving [54]. It is often useful for mental health nurses to ask for these narratives as well: “Those things that you recall were hard to get through. How did you survive them?” Further probes might include “What helped you get through those years since these things happened?” “Were there people who helped you?” [5, 55, 56]. Sometimes constraints to getting through interpersonal violence are sociopolitical, and gender, race, sexual orientation, and religious ideologies may be embedded in trauma narratives. These can be probed after the trauma narratives are told: “Has the trauma you survived led you to consider how race and gender play out in people's lives?” It is often helpful for clients to see that sometimes sociopolitical realities larger than the personal have been a factor in their experiences [57].

## 6. Narrative Embodiment

There are a number of ways in which narratives are experienced and manifested as inscribed on or within the body. A most obvious example is the illumination provided in illness narratives that often focus on the body and bodily experience [50, 58–62]. These stories gain importance when they reflect on present psychiatric symptoms.

### 6.1. Substance Misuse and Recovery Narratives

Common embodied narratives that mental health nurses might elicit are the illness/recovery stories of patients with substance misuse problems, wherein there have been bodily changes related to visible signs of drug abuse, and/or the development of tolerance and withdrawal, for example. It is often lamented that direct questions about drug use may yield unreliable answers because of the stigma associated with addiction, and avoidance of having to stop using drugs and alcohol. Realistically, we know that often treatment is avoided, and that drug use continues over the course of multiple treatment interventions for individual clients. Principles of engaging and maintaining a connection include an imperative to preserve the relationship with clients, whether or not they stop substance misuse and abuse at the current time. We do this in the hope of being available when these clients become more accepting of the need for formal treatment, of joining a mutual help group, or stopping use on their own.

Some narratively based questions that might yield better rapport and open the door to more frank discussions of the substance problem include “How did you come to seek help today? What brought you here at this point in your life? What is happening?” Questions more directly about the substance use itself include “Tell me what happened when you first started using drugs/alcohol?” and “Tell me about a time when using a drug or drink made you feel better.” Clients indirectly reveal the motivations and problems the substances cause in these kinds of stories. Focusing on the values and relationships revealed in stories, versus the substance use itself, is a means of engagement as well as strengthening the nurse-client relationship as the client feels *known at depth* through narratively based communication [58, 63–65]. This ties together the embodied, physiologic processes of habituation with a distinctive view of an individual with a unique past, present, and future.

### 6.2. Scars and Body Art as Narratives

There are other ways that narratives are embodied. Scars from self-harm, such as cutting, can be the focal point for opening a story. Questions might include “What happens when you get into a situation that might lead you to cut?” “Can you tell me a time when you ended up cutting? What stands out about this time for you?” 

Another fascinating way to look for these inscribed narratives is to pay attention to tattoos and body piercings. Often getting a piercing or tattoo marks a significant life event, change, or process. Rather than drawing conclusions about the kind of person who gets tattoos, nurses can inquire about their meaning, “Tell me about how you came to get this tattoo?” and “What about this one?” Males especially, but increasingly women, also, are using tattoos [66, 67]) as a means not only of self-expression about who one is, but also about where one has “traveled,” in their life experiences.

## 7. Accessing Narratives in Client Encounters

Nurses will need to be prepared to elicit and analyze narratives with reference to mental health/illness encounters experienced by individuals. To some extent nurses do this intuitively. In that case, strategies that seem to work in eliciting narratives should be shared, and narrative can become focal, rather than being used only as background information.

### 7.1. Algorithmic Dialogue

Sometimes patients begin relating their initial presenting condition in terms of a story. Too often this story is interrupted because of the need to stay on track in assessing symptoms, with a focus on pathology. Mishler has analyzed the physician's interview as interruptive of narrative. In this case, algorithmic thinking is informed by symptom accounts. This kind of questioning on the part of providers calls for yes or no client responses that are fitted into a decision tree, often also including specific medications to prescribe based on the yes/no answers about symptoms. Because this algorithmic line of questioning is tied to prescribing, advanced practice nurses who prescribe often also use the method in communicating with clients. The algorithmic method of questioning is thought to provide adequate information in the short time frames in which caregivers work. However, if the life narrative is not clearly understood, clinical decisions may be based on erroneous conclusions on the part of the provider, that ultimately cost *more* time and suffering before the right medication or therapy is found [11, 50]. 

We propose that narrative information better contextualizes the client's actual personal and social situation, and helps the caregiver to avoid prescribing on stereotypic notions created by a diagnostic focus. When narrativity is lost, clinicians reason that a symptom cluster indicates a clinical entity, such as bipolar illness, and then treat the illness/disorder, and not the person.

### 7.2. Eliciting Narratives

To elicit narratives in care situations, nurses can open with the question “What has led up to your coming in to the clinic?” Prompts to get narratives versus just descriptive statements or yes/no responses include “What happened?” and “How did that come about?” The focus of narrative is how one has come to the present moment or problem, and thus “how” questions are favored over “why” questions. At the end of a narrative sequence, the provider might ask for an evaluative or outcome question, such as, “How did this affect you?” and “What do you hope will change about this story/situation?” This technique enhances the therapeutic empathy felt by the client, and thus empathy and narrative are not mutually exclusive. Another technique that is especially useful to look into everyday habits and occurrences is to ask about* habitual *narratives as in “What happens in a typical day for you?' or “Tell me what happened yesterday? Is that what you usually do?”.

### 7.3. Exemplars of Narrative and Nonnarrative Approaches

In the following section, some clinical examples regarding the use of narratives with clients are illustrated through quotations from short clinical dialogues recalled from the second author's practice. As previously mentioned, quotations have been edited for conciseness and to avoid including any identifying information. The quotations, in dialogue form, are exemplars showing how approaching the client narratively might change an outcome. The exemplars demonstrate how nurses can step into the narrative space, helping the client to feel known.

In the first case, John is having psychotic experiences of the devil, in the form of a giant lizard.

Nurse: John what happened that brought you here today?
John: I came here against the devil's commands! The devil follows me. And now I am doomed because he wants me dead.
Nurse: How did that happen? Where did you see him?
John: The same as always, I was downtown and he followed me. His tongue is like a lizard's tongue.
Nurse: What happened downtown?
John: I was taking the bus to my mother's house and he made me get off the bus before I got there. He said my mama couldn't save me!
Nurse: Could he find you here?
John: Of course, he has wings and flies through walls.
Nurse: That is a scary story. How will we be able to make him go away?
John: Well the last time I hid under the stairs.
Nurse: What do you think will happen if I gave you some special medication? Might that work to send him away?
John: You don't understand. My mother said I can't come home
Nurse: When were you at home with your mother?
John: Last Tuesday. She said she does not see the devil.
Nurse: Maybe your mother wishes this would go away for you. I can tell by how you described him, and he scares me too. What were you doing?
John: Writing about him in my notebook.
Nurse: I have a notebook I can give you. And some medicine that will keep him quiet. Let's see if that works. Can you take a shot or pill? We would all be safer.
John: Give me a shot.

According to the usual tenets for therapeutic communication, nurses should never agree with clients who are hallucinating or having delusions. But entering the narrative space means becoming part of the story. Some narrative approaches are psychotherapeutically empathetic, but this empathy occurs through co-constructed stories, not only via nurses' analysis of and affirmation of stated feelings on the part of the client. Both approaches strengthen the nurse-client alliance and can be combined.

In the second case, the nurse provides a story, an allegory of sorts. Eliza has severe anxiety and does not believe that medication can help.

Nurse: How does it happen that you get this feeling?
Eliza: Whenever I bump into people I know, my heart starts racing.
Nurse: Did you see someone you know? Where were you?
Eliza: I saw a bunch of people at school. They are all friends from my chemistry class. Not my friends.
Nurse: What happened then?
Eliza: I went into the bathroom and stayed in the stall until I thought they would be gone. I felt like throwing up.
Nurse: There is some medication that might work to help your heart from pounding and could make you more at ease. Let me tell you a story. You know that dogs have a pack order, right? Well one dog, Belle, was constantly afraid of the other dogs, and you know what? Belle would hide and chase her tail. The other dogs just seemed to know Belle was different, and Belle was not in the pack, just always off to the side. Well after seeing Belle's shivering, the vet said medication could help, and when Belle went on medication, she became a part of the pack, because she just acted like the other dogs. It was a happy ending.

Next visit:

Nurse: How has it been?
Eliza: I remembered the story of Belle, and you know I think I have been chasing my tail too. So I started taking the pills. I filled the prescription.
Nurse: What happened?
Eliza: I felt different, but still afraid sometimes. I am sure not one of the pack!
Nurse: Yet?
Eliza: Not yet, I will have to see when it comes to being around people. I want to be part of the pack!

This allegory about dogs was useful because it was not told about another person, such as a previously treated anxious client. If that were the case, it would be easy for the client to reject the story as not being “about me.” The dog displayed some human qualities, and thus was once removed from “real life,” although the story of the dog was real, from the nurse's experience. The idea of a pack made the issue of anxiety as linked to social isolation understandable without the client having to put herself in the place of another person with whom she might not identify.

In a third example, Heidi comes to the clinic often seeking benzodiazepines, on which she has become highly dependent.

Heidi: I need something for my nerves. Just give me a prescription.
Nurse: I cannot do that, Heidi; I won't participate in your addiction.
Heidi: You are a bitch. I can always go somewhere else to get...(alprazolam).
Nurse: I won't participate in your addiction. I can refer you to treatment, because I have a goal that you could someday get along without it, do you think that could also be your goal? What about that?
Heidi: Bitch, all you can do is call me an addict. I am never coming back here!

In this case, a narrative approach was not tried. The client is not able to put her needs into the context of her life, feels labeled, and does not feel known, even though the nurse is consistent in terms of setting limits. We provide an invented dialogue that begins similarly, a dialogue in which a narrative based approach is used to get at the life or self-narrative.

Heidi: I need something for my nerves. Just give me a prescription.
Nurse: What happened to you to upset your nerves?
Heidi: You wouldn't believe me anyway, so what is the use?
Nurse: Well, it would help if you told me about a time when the alprazolam made you feel less nervous.
Heidi: What do you want to hear? How about when I think of having 2 teenage kids and the medication makes me worry less about them.
Nurse: I am sorry that you have trouble with the kids. What have they done that worries you?
Heidi: One has ended up in jail for assault. Is that what you wanna hear?! So give me what I need—a prescription.
Nurse: I cannot write you a prescription, because I, m not sure it will make the situation better in the long run. How do you want this story to end?
Heidi: Bitch, I am never coming back here!

While the outcome of this story is similar to the real case, the addition of a narrative approach provides a context (her children) for her life, and provides for a clearer vision of Heidi as an individual, versus a label that keeps her a flat character, without a story. The second conversation might make Heidi feel known, and she may be surprised at this approach, when her usual experience is like the first exemplar. We believe there would be a greater chance of keeping the nurse-client relationship alive with a narrative approach.

### 7.4. Narrative Therapies

Mental health providers can use narrative therapies [14, 18] as a way to frame the present and future of clients. Psychodynamic therapies are narrative in the degree to which they depend on early life stories to understand a current narrative, revealing problems faced by the client in adulthood. But caregivers, including nurse therapists, can also use narrative to help the individual shape a new self-story, when the question is posed “How would you like your life story to change?” Thus narrative is a flexible entity in the context of a client's life. Narrative can also be embedded in art and music therapies, as in “What is the story behind this picture?” or “this melody?” A complete discussion of the varieties and techniques of narrative therapy is beyond the scope of this paper [see [24, 63, 68]]. We can also envision narrative therapies to reveal family narratives and collective narratives as co-constructed in these groups [21, 47, 69]. Treatment often depends on an ability to conceptualize the remembered narrative as having a temporal and spatial context, reconceptualize the past in the context of the present, and project new narrative turns for a healthier future.

## 8. Narrative versus Direct Communication

Some of what we have said about narrative is sharply in contrast with the foci of conventional therapeutic communication techniques. For example, in narratives information about feelings and meanings are implicit. We can get the information with a few prompts for detail in stories. In contrast, conventional techniques of dialogue in mental health contexts often call for asking “What does that mean to you?” or “How do you feel?” These direct questions are sometimes not culturally appropriate, and may even make clients anxious or annoyed, especially if they have mixed feelings, hard to put into words. 

The beauty of narrative from the perspective of culture is that most, if not all, cultures have a place for storytelling, and narratives are a way of framing everyday experience. Eliciting stories is thus a good way to learn about clients' cultural beliefs, and most importantly, about ways that individuals actually differ *within* a given cultural group. Thus culture as well as individuality within groups is clarified. Stereotyping, whether about gender, ethnicity/race, age, or diagnostic categories is minimized as the narrative contextualizes the other knowledge we have about clients, such as observation data, or psychological testing.

Stories about significant people in the life of a client reveal more about the types of social support and nonsupport that these individuals contribute to the client's life narrative than just the fact of having a partner, close by relative, supportive parents, or having a number of friends or coworkers. What is important from a mental health perspective is what position these other individuals have relative to the client, and what he or she does to be of assistance. “Tell me about a time your brother helped you with your anxiety,” “How did you get to know this friend?”, and “Was there a time when you felt someone else made things worse for you? What happened at that time?” are examples of questions through which social support and interactional data can be gleaned and contextualized by eliciting narratives.

## 9. Implications for Theory and Research

Theory needs to be written about narratives as crucial in the mental health nursing arena so that specifically nursing narrative therapies can be developed. Extant nursing middle range theory concepts such as uncertainty [70, 71], hope [72], and recovery [73] can be adapted more specifically to psychiatric clients' life contexts when they are interpreted through stories. Narrative understandings of subgroups in relation to substance use and misuse, for example, will foster greater awareness among providers of the impact of co-occurring disorders, as well as trauma in early life. The way these elements are interrelated is clarified through stories told to providers. The kind of narrative elicited is important. For instance when stories are developed through 12 step programs, individual distinctions may be concealed rather than highlighted, because similarity and identification among sufferers is emphasized in these groups. These premises need to be studied further for validation.

All types of psychiatric disorders could be better understood through narrative inquiry. Narrative research methods are being refined (1) and adapted to illness and wellness contexts, and will be especially useful in the mental health field because many extant therapeutics depend on the language encounter between client and caregiver.

## 10. Conclusion

Mental health nursing is a field of nursing fraught with ambiguities, and language is predominant in our interventions in many of our clients' cases. This creates a space in which narrative exploration and intervention might permit new understandings of how symptoms correlate with life events. Skilled psychiatric nurses who already elicit narratives will be reaffirmed in these techniques. This exploration of narrative framing of practice provides a new lens through which to know clients at depth and plan with them toward recovery that is in synch with their life situations and their social networks. More purposefully collecting and disseminating narratives about mental health-related cases and events will strengthen the preparation of new generations of mental health nurses who find clinically based narratives to be exciting and vicariously rewarding. 

In the social worlds revealed by narratives, the plot, with its beginning, middle, and ending, takes place in a scene and is carried forward through the characters and actors described. Thus narratives can capture the wholes of experiences that are of central importance to mental health nurses who seek above all to understand the whole person in his/her history and social environment.
